# Autism spectrum disorder-like behaviors induced by hyper-glutamatergic NMDA receptor signaling through hypo-serotonergic 5-HT_1A_ receptor signaling in the prefrontal cortex in mice exposed to prenatal valproic acid

**DOI:** 10.1038/s41386-024-02004-z

**Published:** 2024-10-11

**Authors:** Hitomi Kurahashi, Kazuo Kunisawa, Kenji F. Tanaka, Hisayoshi Kubota, Masaya Hasegawa, Mai Miyachi, Yuka Moriya, Yoichi Hasegawa, Taku Nagai, Kuniaki Saito, Toshitaka Nabeshima, Akihiro Mouri

**Affiliations:** 1 Department of Regulatory Science for Evaluation & Development of Pharmaceuticals & Devices, Fujita Health University Graduate School of Health Science, Aichi, Japan; 2 International Center for Brain Science (ICBS), Fujita Health University, Aichi, Japan; 3 Division of Brain Sciences, Institute for Advanced Medical Research, Keio University School of Medicine, Tokyo, Japan; 4 Division of pharmaceutical science, Faculty of pharmacy, Meijo University, Aichi, Japan; 5 Department of Disease Control and Prevention, Fujita Health University Graduate School of Health Science, Aichi, Japan; 6 Laboratory of Health and Medical Science Innovation (HMSI), Fujita Health University Graduate School of Health Science, Aichi, Japan; 7 Japanese Drug Organization of Appropriate Use and Research, Aichi, Japan

**Keywords:** Autism spectrum disorders, Signalling gradients

## Abstract

Autism spectrum disorder (ASD) is a neurodevelopmental disorder characterized by repetitive behaviors, social deficits, and cognitive impairments. Maternal use of valproic acid (VPA) during pregnancy is associated with an increased risk of ASD in offspring. The prevailing pathophysiological hypothesis for ASD involves excitation/inhibition (E/I) imbalances and serotonergic dysfunction. Here, we investigated the association between glutamatergic-serotonergic neuronal interactions and ASD-like behaviors in mice exposed to prenatal VPA. Prenatal VPA exposure induced excessive repetitive self-grooming behavior and impaired social behavior and object recognition memory in young adult period. Prenatal VPA mice showed hyper-glutamatergic function (increase in basal extracellular glutamate levels and CaMKII phosphorylation) and hypo-serotonergic function (decrease in 5-hydroxyindoleacetic acid and stimulation-induced serotonin [5-HT] release, but an increase in 5-HT transporter expression) in the prefrontal cortex. Treatment with a low-affinity NMDA receptor antagonist (memantine), a selective 5-HT reuptake inhibitor (fluoxetine), and a 5-HT_1A_ receptor agonist (tandospirone) attenuated both the increase in CaMKII phosphorylation and ASD-like behavior of prenatal VPA mice. Opto-genetic activation of the serotonergic neuronal system attenuated impairments in social behavior and object recognition memory in prenatal VPA mice. WAY-100635—a 5-HT_1A_ receptor antagonist—antagonized the effect of fluoxetine on impaired social behavior and object recognition memory. These results suggest that E/I imbalance and ASD-like behavior are associated with hypo-serotonergic receptor signaling through 5-HT_1A_ receptors in prenatal VPA mice.

## Introduction

Autism spectrum disorder (ASD) is a complex neurodevelopmental disorder characterized by stereotyped and repetitive behaviors and/or limited interest (e.g. motor stereotypies, repetitive use of objects, and insistence on sameness of the environment and routines), social communication deficits (e.g. difficulties in expressing emotions, understanding the emotions of others, and empathizing, and the inability to interpret social cues), and cognitive impairment (e.g. deficits in executive functioning, working memory, and episodic memory) [1]. Approximately 1% of the world’s population is affected by ASD [1], with a reported male to female ratio of ~4:1 [2–4]. Valproic acid (VPA) is widely used as an antiepileptic and mood stabilizer. VPA is a teratogen and its administration during pregnancy induces various adverse effects in the fetus [5], increasing the risk of autism in children [6–8]. Based on epidemiological studies, prenatal VPA-exposed rodent models have been developed as ASD-model animals [9, 10].

Excitation/inhibition (E/I) balance and intracellular signaling in neurons play important roles in physiological brain functions [11, 12]. Patients with ASD show that protein expression levels of glutamate receptors and transporters are increased in the brain [13]. Plasma glutamate levels are found to be higher in children with ASD than in healthy controls [14]. The prefrontal cortex (PFC) has been extensively studied to understand the pathophysiology of ASD. Postmortem study shows that number of neuron is increased in the PFC of autism [15]. Magnetic resonance spectroscopy (MRS) study shows increased E/I balance associated with decreased GABA level in the frontal lobe of autism [16]. In the ASD animal model, 15q11-13 duplication mouse exhibit increased the E/I balance in layer 2/3 pyramidal neurons of PFC [17]. Thus, glutamatergic hyper-neurotransmission or GABAergic hypo-neurotransmission in the PFC is associated with ASD pathophysiology, and restoration of the E/I imbalance is thought to be an attractive target for the development of therapeutics.

The serotonergic neuronal system plays an important role in mood, appetite, and behavior, including aggression and sociability [18]. Selective 5-HT reuptake inhibitors (SSRIs) elevate extracellular 5-HT by inhibition of 5-HT transporters (SERT) [19]. It has been reported that SSRIs are effective ASD treatment in adults [20]. There have been randomized controlled trials of SSRIs in children and adolescents with ASD. However, there is no evidence of effect of SSRIs in children and adolescents [21]. The role of the serotonergic system in ASD pathophysiology via E/I imbalances remains unknown. Therefore, this study investigated whether dysfunction of serotonergic-glutamatergic interactions is implicated in the ASD-like behavior of prenatal VPA mice.

## Materials and methods

Please see the supplementary methods for details. A brief description is provided here.

### Animals

The C57BL/6 J mice obtained from Japan SLC (Shizuoka, Japan) and Tph2-tTA::tetO-ChR2(C128S) double transgenic mice were housed in a specific pathogen-free and regulated environment (23 ± 3 °C, and 50 ± 10% humidity). All experiments were performed in accordance with the Regulations for the Management of Laboratory Animals at Fujita Health University.

### Behavioral analysis

Behavioral experiments were performed in a sound-attenuated and air-regulated experimental room, to which the mice were habituated for more than 1 h. The behavioral tests were performed according to the method outlined in a previous report.

### Drugs

On E12.5, pregnant females received a single dose of VPA (*i.p*.). Fluoxetine (FLX), memantine hydrochloride (MEM), tandospirone citrate monohydrate (TAND)were injected intraperitoneally (*i.p*.) 30 min before the behavior test. WAY-100635 maleate (WAY) was injected subcutaneously (*s.c*.) 50 min before the behavior test. The infusion of WAY-100635 into the PFC was performed 30 minutes before the behavior test.

### Real-time reverse transcription-PCR

Total RNA was extracted and converted to cDNA using the Kit. PCRs were performed using the SYBR Green. Expression levels were calculated by the delta-delta Ct method.

### Western blotting analysis

Each protein sample was electrophoresed on SDS-PAGE and subsequently transferred onto a PVDF membrane. The membrane was blocked with skim milk and probed with a primary antibody at 4 °C overnight. The PVDF membranes were washed in TBST, incubated with the appropriate HRP-conjugated secondary antibody, and then washed in TBST. Immunoreactive bands were visualized using imaging system. The band intensities were quantified.

### Monoamine contents

The supernatants of the homogenised brain sample were mixed with 1 M sodium acetate to adjust the pH to 3.0 and injected into an HPLC system equipped with a reversed-phase ODS column and electrochemical detector.

### In vivo micro dialysis

Mice were anesthetized with three types of mixed anesthetic agents before stereotaxic implantation of a guide cannula into the prelimbic PFC. One day after the operation, a micro dialysis probe was inserted through the guide cannula and perfused with artificial CSF. The dialysate was collected and analyzed using HPLC with an electrochemical detector. To stimulate depolarization, Ringer’s solution containing 50 mM KCl was delivered through a dialysis probe for 10 min.

### Opto-genetic manipulations

An optic fiber cannula for micro dialysis and LED cannula for behavioral testing were placed targeting the dorsal raphe nucleus (DRN). During micro dialysis, continuous blue light (470 nm), 5 V light stimulation were repeated for 5 min. A wireless stimulation system was used for opto-genetic behavioral experiments.

### Immunofluorescence

Mice were anesthetized and perfused transcardially with paraformaldehyde (PFA). The postfixed tissues were soaked in sucrose with PBS. The brains were embedded, and cut into sagittal sections using a cryostat. After washing with PBS, sections were blocked with fetal bovine serum in PBS containing Triton-X (PBST), then incubated with primary antibodies in PBS at 4 °C overnight. The sections were incubated with secondary antibodies and Hoechst 33342. Sections were visualized under a confocal laser microscope.

### Statistical analysis

All results were expressed as the mean ± SEM for each group. Differences between groups were analyzed using one- or two-way ANOVA, followed by Tukey’s multiple-comparison test. Post hoc tests were performed when F in ANOVA achieved the necessary level of statistical significance (*p* < 0.05) and there was no significant variance inhomogeneity. Student’s *t*-tests were used to compare the two sets of data. Detailed statistical results for each analysis are listed in Supplementary Table 2.

## Results

### ASD-like behavior in prenatal VPA mice

**Fig. 1:**
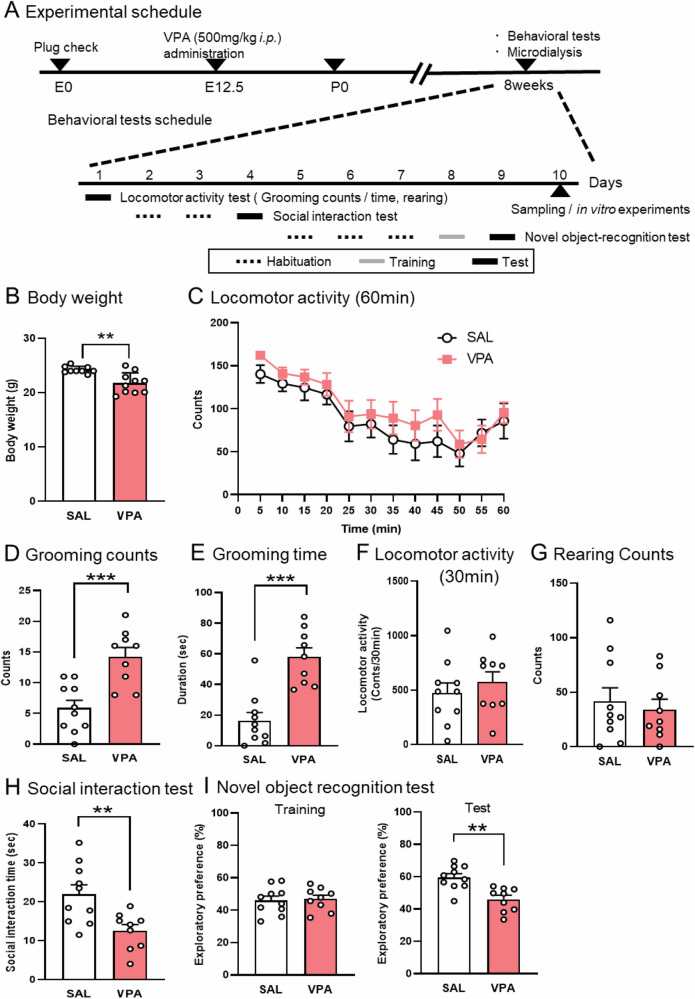
ASD-like behavior in the male prenatal VPA mice. Experimental schedule (**A**). Male prenatal VPA mice were measured body weight at 8 weeks old (**B**). Locomotor activity test (**C**–**G**): Prenatal VPA mice were placed in a novel environment, and locomotor activity was evaluated every 5 min for 60 min (**C**). Repetitive behavior was assessed by counting grooming behavior (**D**) and duration of grooming behavior (**E**) in the latter half of the test (31–60 min) for 30 min. Locomotor activity (**F**) and vertical activity were assessed by counting rearing behavior (**G**) in the latter half of the test. Social interaction test (**H**): Prenatal VPA mice were placed in an apparatus with an unfamiliar mouse, and social behavior was evaluated for 10 min. Novel object recognition test (**I**): Prenatal VPA mice were placed in an apparatus containing two objects during the training session. After 24 h, the mice were placed back into the apparatus with novel and familiar objects during the test session. Cognitive function was assessed as the ratio of the amount of time spent exploring any one of the two objects (training session) to the novel object (retention session). Each column represents the mean ± SEM. **p < 0.01, ***p < 0.001 versus control mice (SAL; saline). *n* = 9–10/ each group.

There is growing recognition that ASD is a lifelong neurodevelopmental condition that can affect functioning throughout adulthood [22]. While ASD affects both males and females, sex-specific phenotypic characterization could make it much more commonly diagnosed in males [23]. To evaluate the face validity of male and female prenatal VPA mice as an ASD model, they were subjected to behavioral tests at adulthood (8 weeks old) (Fig. 1A). Prenatal VPA exposure delayed physical development [24, 25]. Both male and female prenatal VPA mice showed lower body weight at 8 weeks old (Fig. 1B and Supplementary Fig. 1A). In the locomotor activity test for 60 min, both male and female groups initially displayed an increase in exploratory activity in a novel environment, and the activity decreased with habituation to the environment (Fig. 1C and Supplementary Fig. 1B).

To evaluate repetitive behaviors, we measured the duration and count of grooming in the latter part (31–60 min) for 30 min. The duration and count of grooming were increased in the male but not female prenatal VPA mice (Fig. 1D, E and Supplementary Fig. 1C and D). There was no difference in locomotor activity and number of rearing as an index of vertical activity (Fig. 1F, G and Supplementary Fig. 1E and F).

Next, we investigated whether social behavior decreased as a behavioral model of social deficits. In the social interaction test, the male but not female prenatal VPA mice showed a decrease in social interaction time, suggesting a social impairment (Fig. 1H and Supplementary Fig. 1G).

Third, we investigated whether cognitive impairment was observed in the prenatal VPA mice using a novel object-recognition test. In the retention session, the exploratory preference for a novel object in the male but not female prenatal VPA mice was decreased, suggesting impairment of object-recognition memory (Fig. 1I and Supplementary Fig. 1H).

These results suggest that the male rather than female prenatal VPA mice showed face validity in ASD models, such as ASD-like behavior.

In addition, the alternation behavior in the Y-maze test, the duration in the central zone of the open field test, and the duration in the open arms of the elevated plus maze test were not altered in the prenatal male VPA mice (Supplementary Fig. 2A–C). These data suggest that executive function and anxiety were not apparently altered in the prenatal male VPA mice.

### Hyper-glutamatergic function in the prefrontal cortex of prenatal VPA mice

**Fig. 2:**
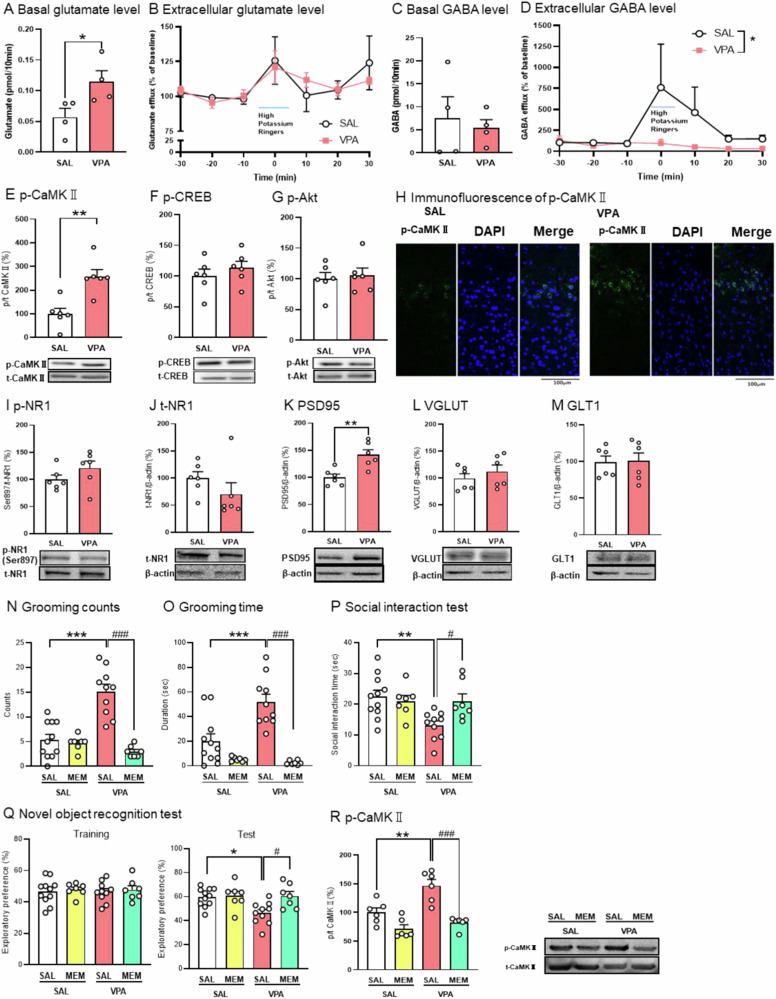
Hyper-glutamatergic function in the prefrontal cortex. Extracellular levels of glutamate and GABA (**A**–**D**): Basal extracellular level of glutamate (**A**) and GABA (**C**), and high potassium-stimulated release of glutamate (**B**) and GABA (**D**) in the prefrontal cortex (PFC) of prenatal VPA mice were determined by micro dialysis. Protein expression of phosphorylated CaMKII (**E**), phosphorylated CREB (**F**), and phosphorylated Akt (**G**) in the PFC as determined by western blotting. Immunofluorescence of phosphorylated CaMKII in the PFC of control and VPA-exposed mice (**H**). The protein levels of phosphorylated NR1 (**I**), total NR1 (**J**), PSD95 (**K**), phosphorylated VGLUT (**L**) and phosphorylated GLT1 (**M**) in the PFC. Behavioral test (**N**–**Q**): Memantine (MEM: 10 mg/kg, *i.p*.) was administered to prenatal VPA mice 30 min before the measurement of counts (**N**) and duration (**O**) of grooming behavior in the latter half of the test (31–60 min) for 30 min, social interaction test (**P**), and novel object recognition test (**Q**). Phosphorylation ratio of CaMKII in the PFC was determined by western blotting (**R**). PFC was extracted 30 min after MEM administration. Values represent the mean ± SEM. *p < 0.05, **p < 0.01 and ***p < 0.001 versus control mice. ^#^*p* < 0.05 and ^###^*p* < 0.001 vs. saline-treatment VPA-exposed mice. (**A**–**D**) *n* = 4; (**E**–**G**), (**I**–**M**), (**R**) *n* = 6; (**H**) *n* = 3; (**N**–**Q**) *n* = 7–11/ each group.

Based on the E/I imbalance in the pathophysiological hypothesis of ASD [26, 27], we first measured extracellular glutamate levels in the prelimbic PFC of prenatal VPA mice using in vivo micro dialysis. The basal levels of extracellular glutamate in these mice were higher (Fig. 2A), but there was no difference in the response ratio to high potassium stimulation (50 mM) (Fig. 2B). Although there was no difference in the basal level of extracellular GABA (Fig. 2C), the response to high potassium stimulation was lower in the prenatal VPA mice (Fig. 2D).

Next, we investigated whether higher basal extracellular glutamate levels activate postsynaptic glutamatergic signaling in the PFC. Calcium/calmodulin-dependent protein kinase II (CaMKII) is activated by Ca^2+^ entry through the NMDA receptors [28]. The ratio of phosphorylated/total CaMKII increased in the PFC of VPA mice (Fig. 2E). However, the phosphorylation levels of CREB and Akt were not different (Fig. 2F, G). To investigate the localization of enhanced CaMKII phosphorylation in the prelimbic PFC of prenatal VPA mice, we performed immunohistochemistry. Phosphorylated CaMKII was highly expressed in the 2/3 layer of the PFC in prenatal VPA mice (Fig. 2H).

Enhanced NMDA receptor signaling may be due to enhanced expression and phosphorylation of NMDA receptors [29]. The ratio of phosho-NR1 at ser897/ total NR1 and the expression level of total NR1 are comparable (Fig. 2I, J).

Next, we investigated whether the overexpression of presynaptic and postsynaptic proteins modulate glutaminergic neurotransmission and receptor signaling. Postsynaptic density protein 95 (PSD95) was increased in the PFC of prenatal VPA mice (Fig. 2K). There were no differences in the protein levels of VGLUT and GLT1 (Fig. 2L, M). These results suggest that there is prenatal hyper-glutamatergic function and signaling in the PFC of VPA mice.

To investigate whether hyper-glutamatergic function is involved in ASD-like behavior of prenatal VPA mice, they were administered MEM (10 mg/kg, *i*.*p*.), a low-affinity voltage-dependent uncompetitive NMDA receptor antagonist [30]. MEM attenuated all of the ASD-like behaviors (Fig. 2N–Q). Furthermore, MEM attenuated the enhancement of CaMKII phosphorylation in the PFC of prenatal VPA mice (Fig. 2R). These results suggest that MEM attenuates ASD-like behaviors, by suppressing hyper-glutaminergic signaling in the PFC of prenatal VPA mice.

### Hypo-serotonergic function in the prefrontal cortex (PFC) of prenatal VPA mice

**Fig. 3:**
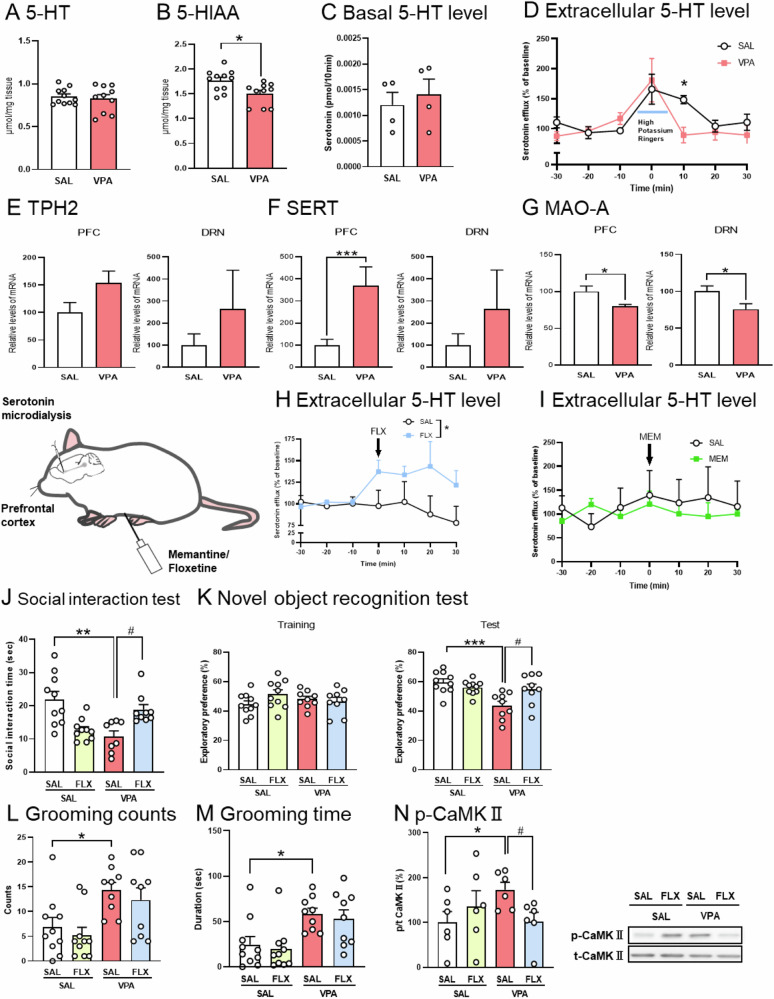
Hypo-serotonergic function in the prefrontal cortex (PFC) of prenatal VPA mice. Serotonin (5-HT) and its metabolite contents: 5-HT (**A**) and 5-HIAA (**B**) in the prefrontal cortex (PFC) were measured by HPLC. Extracellular levels of 5-HT by stimulation of high potassium: The basal level (**C**) and the level of extracellular (**D**) 5-HT in the PFC of prenatal VPA mice were measured by stimulation of high potassium. The mRNA expressions of TPH2 (**E**), serotonin transporter (SERT) (**F**) and monoamine oxidase-A (MAO-A) (**G**) in the PFC and dorsal raphe nucleus (DRN) of prenatal VPA mice were measured by real-time reverse transcription- PCR. Extracellular serotonin (5-HT) levels in the prefrontal cortex (PFC) after Fluoxetine (FLX) (**H**) and Memantine (MEM) (**I**) administration. Behavioral tests: Fluoxetine (FLX: 10 mg/kg, *i.p*.) was administered to prenatally exposed VPA mice 30 min before the behavioral tests. social interaction test (**J**), novel object recognition test (**K**), and count (**L**) and duration (**M**) of grooming behavior. The phosphorylation ratio of CaMKII in the PFC was measured using western blotting (**N**). Values represent the mean ± SEM. *p < 0.05, **p < 0.01, and ***p < 0.001 versus control mice. ^#^*p* < 0.05, versus saline-treated VPA-exposed mice. (**A**, **B**, **E**–**G**) *n* = 10–11; (**C**, **D**) *n* = 4; (**H**) *n* = 3; (**I**) *n* = 5; (**J**–**M**) *n* = 9–10; (**N**) *n* = 6/each group.

Serotonin (5-HT) is implicated in mood, sleep, obsessive-compulsive symptoms, and other comorbidities in ASD [31]. In the PFC, 5-HT content did not differ (Fig. 3A). The content of the 5-HT metabolite (5-hydroxyindoleacetic acid: 5-HIAA) in the PFC of prenatal VPA mice was lower (Fig. 3B). To further investigate serotonergic function in prenatal VPA mice, we measured extracellular 5-HT levels using in vivo micro dialysis. There was no difference in the basal level of extracellular 5-HT (Fig. 3C). The amount of extracellular 5-HT was increased by stimulation with high potassium (50 mM) in the PFC, but the level was decreased in the prenatal VPA mice (Fig. 3D).

The dysfunction was selective in the serotonergic neurons of the PFC, since there was no difference in other monoamines or their metabolites and in any monoamines or their metabolites in the striatum, hippocampus, or amygdala (Supplementary Table.1).

It is possible that prenatal VPA exposure affects the expression of serotonergic neuronal system-related proteins such as synthetases, degradation enzymes, and transporters. Tryptophan hydroxylase type 2 (TPH2) is an important enzyme for the synthesis of 5-HT from tryptophan [32, 33]. In the mRNA level of TPH2, there was no difference in the PFC and DRN of prenatal VPA mice (Fig. 3E). SERT is a protein present in the synaptic cleft that regulates the concentration of 5-HT. The mRNA level of SERT was increased in the PFC of prenatal VPA mice, but not in the DRN. (Fig. 3F). Monoamine oxidase (MAO-A) is an important enzyme that metabolizes 5-HT. The mRNA level of MAO-A decreased y in the PFC and DRN of prenatal VPA mice (Fig. 3G). These results suggest that prenatal VPA exposure induces hypo-serotonergic function in the PFC by decreasing the 5-HT content in the synaptic cleft by increasing SERT expression.

SSRIs inhibit the reuptake of 5-HT by blocking SERT, thereby increasing extracellular 5-HT levels. To investigate the relationship between ASD-like behaviors and hypo serotonergic function in prenatal VPA mice, the mice were administered FLX (20 mg/kg, *i.p*.) and subjected to in vivo micro dialysis and behavioral tests. In vivo micro dialysis revealed that FLX increased extracellular 5-HT levels in the PFC of prenatal VPA mice (Fig. 3H). We investigated whether the serotonin release was associated with the attenuating effect of MEM. MEM did not affect extracellular serotonin levels in the PFC of prenatal VPA mice (Fig. 3I).

FLX attenuated decreases in social interaction time (Fig. 3J) and exploratory preferences (Fig. 3K) in prenatal VPA mice.

FLX did not attenuate an increase in grooming counts or duration (Fig. 3L, M).

These results suggest that activation of 5-HT transmission by FLX attenuates the impairment of sociability and object recognition memory in prenatal VPA mice. It is possible that FLX attenuates ASD-like behavior by increasing 5-HT levels. Furthermore, CaMKII phosphorylation indicated that FLX attenuated the enhancement of CaMKII phosphorylation in the PFC of these mice (Fig. 3N). These data suggest that FLX improves social and cognitive deficits, but not repetitive behavior, by suppressing hyper-glutaminergic neurotransmission and signaling by increasing 5-HT levels in the PFC of prenatal VPA mice.

### Optogenetic activation of the dorsal raphe serotonergic neurons attenuates social and cognitive deficits in prenatal VPA mice

**Fig. 4:**
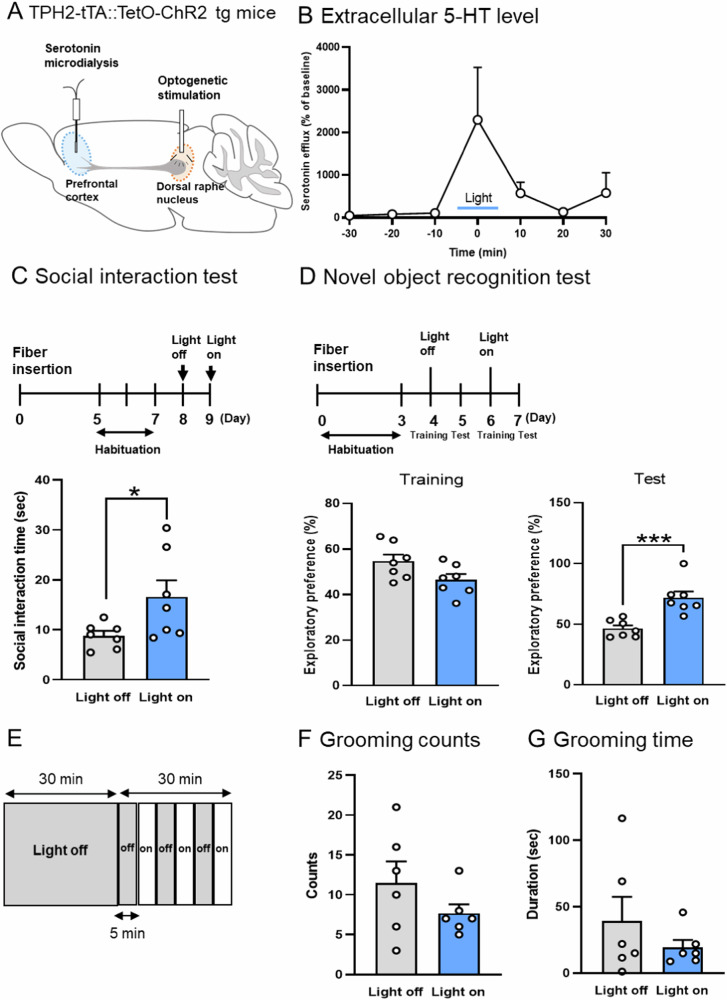
Optogenetic activation of the dorsal raphe serotonergic neurons attenuates social and cognitive deficits in prenatal VPA mice. Optic probe and micro dialysis probe were implanted in the dorsal raphe nucleus (DRN) and prefrontal cortex (PFC). The light was radiated for 5 min (**A**). Extracellular serotonin levels in the PFC by opto-genetic stimulation in the DRN (**B**). Behavior tests under opto-genetics (**C**–**G**): Social interaction time of prenatal VPA mice in light off and on conditions (**C**). Cognitive function of prenatal VPA mice in novel object recognition test. Exploratory preference was measured during a 10 min session. Light stimulation was performed in training session (**D**). The light stimulation was delivered in the period of 5 min for 30 min (**E**). Counts and duration of grooming behavior were measured and those in the light “on” were compared to light “off” conditions in prenatal VPA mice (**F**) (**G**). Values represent the mean ± SEM. *p < 0.05 and ***p < 0.001 versus prenatal VPA mice with non-light stimulation. *n* = 6–7/each group.

To investigate whether ASD-like behavior in prenatal VPA mice is attenuated by the activation of DRN, we implanted an optical fiber in the DRN of TPH2-tTA::TetO-ChR2 tg mice, which is specifically expressed ChR2 in serotonergic neurons (Fig. 4A) and performed in vivo micro dialysis and behavioral tests. Light stimulation of the DRN increased extracellular 5-HT levels in the PFC of prenatal VPA mice (Fig. 4B).

The social interaction time under light stimulation was higher in the prenatal VPA mice (Fig. 4C). In the novel object recognition test, training and retention sessions were conducted under non-stimulation conditions after three days of habituation. The object recognition memory in mice trained under light stimulation was higher (Fig. 4D).

In the locomotor activity test, we measured the duration and count of grooming in the latter half of the test (31–60 min) for 30 min. Light stimulation was turned on and off every 5 min for 30 min (Fig. 4E). Grooming counts and duration under light stimulation were not different (Fig. 4F, G). These results suggest that the opto-genetic activation of dorsal raphe serotonergic neurons attenuates social and cognitive deficits, but not repetitive behavior, in prenatal VPA mice.

### 5-HT_1A_ receptor involvement in fluoxetine attenuative effects on social and cognitive deficits in the prenatal VPA mice

**Fig. 5:**
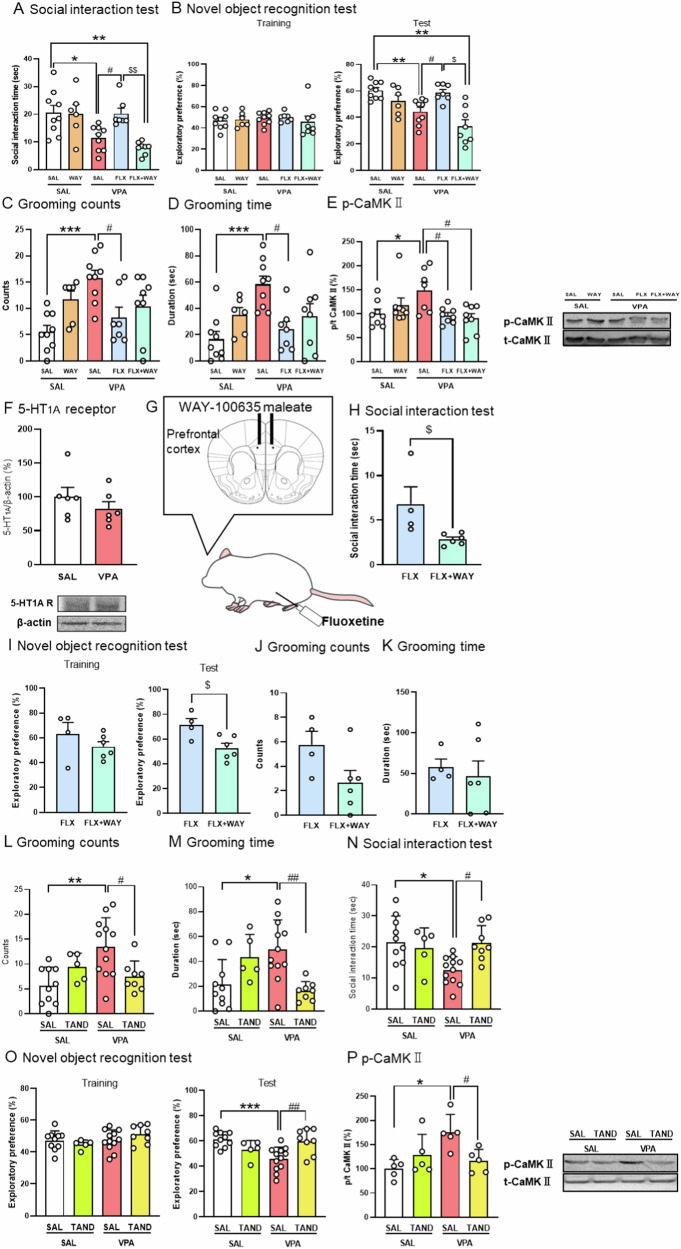
5-HT_1A_ receptor involvement in fluoxetine attenuative effects on social and cognitive deficits in the prenatal VPA mice. WAY-100635 (WAY:0.3 mg/kg, *s.c*.) and Fluoxetine (FLX: 20 mg/kg, *i.p*.) were administrated to prenatal VPA mice 50 min and 20 min, respectively before the social interaction test (**A**), novel object recognition test (**B**), the measurement of counts (**C**) and duration (**D**) of grooming behavior in the latter half part (31–60 min) of test for 30 min. The phosphorylation ratio of CaMK II in the PFC was measured by Western blotting (**E**). The protein expression of 5-HT_1A_ receptor in the PFC of prenatal VPA mice (**F**). Bilateral infusion of WAY (10.0 μg/0.2 μl) into PFC and systemic administration of FLX (20 mg/kg, *i.p*.) to prenatal VPA mice (**G**) were performed 30 min before social interaction test (**H**), novel object recognition test (**I**), and the measurement of counts (**J**) and duration (**K**) of grooming behavior. Tandospirone (TAND: 0.1 mg/kg, *i.p*.) was administered to prenatally exposed VPA mice 30 min before the measurement of counts (**L**) and duration (**M**) of grooming behavior in the latter half part (31–60 min) for 30 min, social interaction test (**N**), and novel object recognition test (**O**). Expression of p-CaMKII in the PFC (**P**). Values represent the mean ± SEM. *p < 0.05, **p < 0.01, and ***p < 0.001 versus control mice. ^#^*p* < 0.05, and ^##^*p* < 0.01 versus saline-treated VPA-exposed mice. ^$^*p* < 0.05, and ^$$^*p* < 0.01, versus FLX-treated VPA-exposed mice. (**A**–**F**) *n* = 6–9; (**H**–**K**) *n* = 4–6; (**L**–**O**) *n* = 5–12; (**P**) *n* = 5/each group.

5-HT_1A_ receptors are expressed on glutamatergic neurons and negatively control glutamate release [34, 35]. In clinical studies, a 5-HT_1A_ partial agonist, buspirone, has potential effects on various symptoms of ASD, particularly anxiety [36, 37]. The 5-HT_1A_ receptors could be involved in the pathogenesis of ASD and could be a potential therapeutic target. To investigate the involvement of 5-HT_1A_ receptors in the attenuative effect of FLX on social and cognitive deficits, prenatal VPA mice were co-administered WAY (0.3 mg/kg, s.c.), a 5-HT_1A_ receptor antagonist, and FLX and subjected to behavioral tests. WAY antagonized the attenuating effect of FLX on the decreased social interaction time of the mice (Fig. 5A). Further, WAY antagonized the attenuating effect of FLX on the decreased exploratory time to novel objects of the mice (Fig. 5B), but failed to antagonize the attenuating effect of FLX on the increased grooming counts and duration (Fig. 5C, D). When we performed western blotting for CaMKII phosphorylation, the WAY failed to antagonize the attenuating effect of FLX on the enhancement of CaMKII phosphorylation in the PFC of prenatal VPA mice (Fig. 5E). There was no difference in the protein expression of 5-HT_1A_ receptors (Fig. 5F). To investigate the involvement of prefrontal cortical 5-HT_1A_ receptors in the attenuating effect of FLX, prenatal VPA mice were bilaterally infused with WAY (10.0 μg/ 0.2 μl) into the PFC, and systemically administered with FLX and subjected to behavioral tests (Fig. 5G). Microinfusion of WAY into PFC antagonized the attenuating effect of FLX on the decreased social interaction time of the mice (Fig. 5H), decreased exploratory time to novel objects of the mice (Fig. 5I), but failed to antagonize the attenuating effect of FLX on the increased grooming counts and duration (Fig. 5J, K). WAY did not affect their behavior in control mice (Supplementary Fig. 3A–D). These results suggest that FLX attenuates social and cognitive deficits, but not repetitive grooming behavior, in prenatal VPA mice by activating prefrontal cortical 5-HT_1A_ receptors.

To investigate whether direct activation of 5-HT_1A_ receptors attenuates ASD-like behaviors and hyper-glutamatergic function, prenatal VPA mice were administered TAND (0.1 mg/kg, *i.p*.)a 5-HT_1A_ receptor agonist-and subjected to behavioral tests. TAND attenuated increases in grooming counts and duration (Fig. 5L, M). It also attenuated decreases in social interaction time (Fig. 5N) and decreases in exploratory preference for novel objects (Fig. 5O). Furthermore, TAND attenuated the enhancement of CaMKII phosphorylation in the PFC (Fig. 5P). These results suggest that TAND activates 5-HT_1A_ receptor selectively and attenuates all abnormal behaviors-repetitive, social, and cognitive-by suppressing hyper-glutaminergic signaling in the PFC of prenatal VPA mice.

## Discussion

Here, we investigated the involvement of glutamatergic-serotonergic neuronal interactions in eliciting ASD-like behavior in prenatal VPA mice. In this study, prenatal VPA mice showed increase of grooming behaviors, decrease of social behavior, decrease of exploratory preference in males but not females (Fig. 1 and Supplementary Fig. 1). These sex-dependent ASD-like behaviors in the prenatal VPA mice are consisted with previous reports [38, 39]. An E/I imbalance in the PFC is involved in ASD pathophysiology [40–42]. Here, the basal levels of extracellular glutamate and phosphorylation of CaMKII were elevated in the prelimbic PFC of prenatal VPA mice (Fig. 2). CaMKII activation is triggered by Ca^2+^ influx through NMDA receptors [43]. The PFC consists of a laminar structure with principal excitatory neurons primarily located in layers 2/3, 5, and 6 [44]. Layer 2/3 pyramidal neurons are implicated in social behavior and cognitive function [45, 46], and an increased number of these neurons has been reported in patients with ASD [47]. Layer 2/3 neurons in the PFC receiving projection from thalamus [48] and hyper-connectivity between PFC and thalamus is observed in ASD [49]. Here, our immunohistochemistry data showed that phosphorylated CaMKII-positive cells were predominantly observed and increased around layer 2/3 of the prelimbic PFC of prenatal VPA mice. These results suggest that the localization of layer2/3 specific hyper-glutamatergic signaling in the PFC of these mice, presumably due to receiving projections from the thalamus, shows construct validity, such as the anatomical features observed in patients with ASD. This suggests that the expression of proteins regulating the glutamatergic system is increased in the PFC. However, our western blotting data showed that there were no changes in the protein levels of phosphorylated NR1 at serine 897 residue, and total NR1, VGLUT, and GLT1 in the PFC (Fig. 2). Our results suggest that hyper-glutamatergic neurotransmission and signaling in prenatal VPA mice are likely not associated with glutamatergic protein expression levels.

MEM may be beneficial for the treatment of ASD-associated irritability [50]. But systematic review reports that MEM is largely negative for core symptoms of ASD [51, 52]. Here, MEM attenuated not only repetitive behaviors and social and cognitive deficits, but also increased phosphorylated CaMKII in the PFC of prenatal VPA mice (Fig. 2). The electrophysiological analysis enhanced synaptic plasticity in the PFC of prenatal VPA mice [53]. Although further experiments using electrophysiological technics are needed, these data suggest that hyper-glutamatergic neurotransmission and signaling in the PFC are involved in ASD-like behavior in these mice, which are consisted with previous electrophysiological report [53].

5-HT plays an important role in brain development [54]. In patients with ASD, lower 5-HT levels have been observed in several brain regions, including the PFC and DRN [55]. Here, prenatal VPA mice showed decreased tissue contents of the metabolite, 5-HIAA, in the PFC. In vivo micro dialysis data also showed that the evoked extracellular 5-HT levels following high-potassium stimulation were decreased. In addition, SERT mRNA levels were increased in the PFC of prenatal VPA mice (Fig. 3). These data suggest that these mice show serotonergic hypofunction-such as a decrease in stimulation-induced 5-HT release and 5-HT in the synaptic cleft-due to 5-HT reuptake increases through SERT level increases.

Our pharmacological approach showed that FLX attenuated the social and cognitive deficits in prenatal VPA mice (Fig. 3). The DRNs—origin of the major ascending serotonergic neurons in the forebrain—projects specifically to the PFC and plays an important role in cognitive and emotional functions [56]. Furthermore, reduced 5-HT levels and DRN volumes are associated with ASD [57, 58].

Opto-genetic activation of serotonergic neurons in the DRN of prenatal VPA mice strongly increased extracellular 5-HT levels in the PFC and attenuated social and cognitive deficits (Fig. 4). These data suggest that activation of serotonergic neurons in the DRN projecting to the PFC attenuates ASD-like behavior in these mice.

MAO-A catalyzes the oxidative deamination of 5-HT into its corresponding aldehyde, which is converted to 5-HIAA. Thus, 5-HIAA levels in the brain reflect MAO-A activity. The mRNA level of MAO-A was decreased in prenatal VPA mice (Fig. 3). However, it is unlikely that a decrease in MAO-A induces the downregulation of 5-HT metabolism in 5-HIAA. Because in vivo micro dialysis and pharmacological and opto-genetic data suggest a decrease in serotonergic function in the PFC of prenatal VPA mice (Figs. 3 and 4), it is possible that the MAO-A decrease in these mice is due to a substrate (5-HT) decrease.

Among 5-HT receptors, 5-HT_1A_ has the highest affinity for serotonin [59]. 5-HT_1A_ receptors are expressed in glutamatergic neurons and suppress glutamate release [34]. Here, we investigated the association between hypo-serotonergic and hyper-glutamatergic neurotransmission in the PFC of prenatal VPA mice. The 5-HT_1A_ receptor antagonist, WAY, blocked the attenuative effect of FLX on social and cognitive deficits (Fig. 5). FLX but not MEM increased extracellular 5-HT levels in the PFC. These results suggest that FLX activates 5-HT_1A_ receptors and attenuates social and cognitive deficits in these mice by suppressing glutamate release. Activation of 5-HT_2A_ receptors suppresses voltage-dependent Ca^2+^ channel and NMDA receptor mediated signaling in the PFC [60]. It is possible that FLX over increases 5-HT levels which activates 5-HT_2A_ as well as 5-HT_1A_ receptors and suppresses Ca^2+^ signaling. Pharmacological (FLX) and opto-genetic activation of serotonergic neurons in the PFC attenuates social and cognitive deficits, but not repetitive behavior. However, the 5-HT_1A_ receptor agonist (TAND) attenuates not only repetitive behavior but also social and cognitive deficits and hyper-glutamatergic signaling in the PFC of prenatal VPA mice. The 5-HT_2B_ and 5-HT_2C_ receptors are involved in repetitive behavior, and their antagonists reduce grooming frequency [61, 62]. Like FLX, opto-genetic stimulation activates serotonergic neurons, but not receptor-specifically; thus, it failed to decrease the grooming count and duration. Although the efficacy of opto-genetic stimulation of the 5-HT neuron terminals and 5-HT receptor subtype-positive neurons in the PFC are needed as future experiments, these data suggest that pharmacological and opto-genetic activation of serotonergic neurons attenuates social and cognitive deficits and hyper-glutamatergic signaling in the PFC of prenatal VPA mice via 5-HT_1A_ receptors but fails to attenuate repetitive behavior by simultaneous activation of 5-HT_1A_ receptors. Therefore, these results suggest that 5-HT_1A_ receptors play important roles in hypo-serotonergic and hyper-glutamatergic interactions in the PFC and in ASD-like behavior in prenatal VPA mice.

## Conclusion

ASD-like behaviors are induced by the hypo-serotonergic function in 5-HT_1A_ receptor signaling through the overexpression of SERT, which is associated with hyper-glutamatergic neurotransmission and signaling in prenatal VPA mice.

## Supplementary information

Supplemental information

Supplemental Table 2

## Data Availability

The datasets are available from the corresponding author upon reasonable request.
