# Supplementary material for: Autism spectrum disorder-like behaviors induced by hyper-glutamatergic NMDA receptor signaling through hypo-serotonergic 5-HT1A receptor signaling in the prefrontal cortex in mice exposed to prenatal valproic acid
Source: Neuropsychopharmacology. 2024 Oct 11;50(5):739–50. doi: 10.1038/s41386-024-02004-z (PMC11914464; doi:10.1038/s41386-024-02004-z)
Supplement: Supplementary file 1 — Supplemental information [file 41386_2024_2004_MOESM1_ESM.pdf]

Supplemental information

Supplemental materials and methods

1. Animals

All mice were mature males aged 8 weeks. The C57BL/6J mice were obtained from Japan SLC (Shizuoka, Japan). Tph2-tTA::tetO-ChR2(C128S) double transgenic mice [1] were backcrossed with the C57BL/6N strain. Genotyping protocol was described previously [1]. The mice were housed in specific pathogen-free environments within our animal facility prior to use and were kept in a regulated environment (23 ± 3 °C, and 50 ± 10 % humidity), with a 12-h light/dark cycle (lights on at 8:00 A.M., off at 8:00 P.M.). Food from the MF Oriental Yeast Co. Ltd., (Tokyo, Japan) and tap water were provided ad libitum. All experiments were performed in accordance with the Regulations for the Management of Laboratory Animals at Fujita Health University.

2. Prenatal VPA treatment

Female mice aged 6 to 7 weeks were mated with male mice of the same age in the evening and checked for vaginal plugs the following morning. Female mice with vaginal plugs were considered embryonic day 0 (E0). On E12.5, pregnant females received a single dose of VPA sodium salt (Tokyo Chemical Industry, Tokyo, Japan) at a dose of 500 mg/kg (*i.p.*) dissolved in saline. The control group was administered saline alone. Eight-week-old male mice were used as the ASD-model mice and control mice because the male mice has been reported to show severe ASD-like behavioral abnormalities [2].

3. Behavioral analysis

All behavioral tests were performed between 10:00 A.M. and 6:00 P.M. Behavioral experiments were performed in a sound-attenuated and air-regulated experimental room, to which the mice were habituated for more than 1 h.

3.1 Spontaneous locomotor activity test

The locomotor activity test was performed according to the method outlined in a previous report [3]. Each mouse was placed in an acrylic box (30 × 30 × 30 cm) with a brightness of 10 lx. Locomotion in the novel environment was measured every 5 min for 60 min using an infrared sensor (MDC System; Brain Science Idea, Osaka, Japan). The frequency and duration of rearing and grooming events were counted for 30 min between 31 to 60 min for the 1 h measurement.

3.2 Social interaction test

Social interaction test was performed according to the method outlined in previous reports [3] [4]. The apparatus consisted of a square open arena with gray walls (25 × 25 × 30 cm). Light was diffused to minimize shadows in the arena and maintained at 10 lx. Each mouse was placed alone in the test box for 10 min on two consecutive days before the social interaction test (habituation). On the test day, each mouse was randomly assigned to the same gender and age C57BL/6J mice who were used as unfamiliar partners. The mice and unfamiliar partners were placed in a box for 10 min. The duration of social interactions—sniffing, grooming, following, mounting, and crawling, but not aggressive behavior—was recorded, and the total social interaction time was measured.

3.3 Novel-object recognition test

The novel-object recognition test was performed according to methods outlined in

previous reports [5] [6]. The test consisted of three sessions: habituation, training, and retention. Each mouse was individually habituated to a Plexiglas box (30 × 30 × 35 cm) through a 10 min exploration time without any objects present for 3 days (habituation session). Light was diffused to minimize shadows in the arena and maintained at 10 lx. During the training session, two various objects—wooden triangular prism, and square pyramids, which were different in shape and color but similar in size—were placed in the back corner of the box. A mouse was then placed midway toward the front of the box and the total time spent exploring the two objects was recorded for 10 min. The object exploration behavior of the mouse was defined as either having its head facing the object or touching/sniffing it. During the retention session, the mouse was placed back in the same box 24 h after the training session, but one of the familiar objects used during training was replaced with a novel object- a golf ball. The mice were then allowed to explore freely for 10 min, and the time spent exploring each object was recorded. A preference index, the ratio of the amount of time spent exploring any one of the two objects (training session) or the novel object (test session) to the total time spent exploring both objects, was used to measure cognitive function.

3.4 Elevated plus-maze test

Elevated plus-maze test was performed according to the method outlined in previous reports [3] with minor modifications. The elevated plus-maze consisted of two open (25 x 8 x 0.5 cm) and two closed (25 x 8 x 20 cm) arms extending from a common central platform (8 x8 cm) to form a plus shape. The entire apparatus was elevated to a height of 50 cm above floor level with a brightness of 50 lx. The test was started by placing a mice on the central platform of the maze facing an open arm. The time spent in each of the

open arms and closed arms were measured for 5 min using images captured on video the ANY-maze video tracking system (Stoelting Co., Ltd., Wood Dale, IL, USA).

3.5 Open field test

Elevated plus-maze test was performed according to the method outlined in previous reports [3] with minor modifications. The open field consisted of a square area (34 cm × 34 cm × 24 cm) set in a dark room. A light (100lx) was positioned 100 cm above the center of the floor. Each mouse was placed in a section of the corner zone, and then allowed to freely explore its environment. The amount of time spent in each zone (corner zone, center zone) was measured for 5 min using the ANY-maze video tracking system (Stoelting Co., Ltd).

3.6 Y-maze test

The Y-maze test was performed according to the method outlined in previous reports [3] with minor modifications. The maze was made of gray plastic; each arm was 40 cm long, 12 cm high, 3 cm wide at the bottom, and 10 cm wide at the top. The arms converged at an equilateral triangular that was 4 cm at its longest axis. Each mouse was placed at the center of the apparatus and allowed to move freely through the maze during an 8-min session. Arm entries were recorded by video camera. Alternation was defined as successive entry into the three different arms, counting overlapping triplet sets. Alternation behavior (%) was calculated as the ratio of actual alternations to possible alternations, defined as (number of arm entries – 2) × 100.

4. Drugs

Fluoxetine (FLX; Tokyo Chemical Industry)(20 mg/kg), memantine hydrochloride (MEM; Tokyo Chemical Industry) (10 mg/kg), tandospirone citrate monohydrate (TAND; Tokyo Chemical Industry) (0.1 mg/kg) and WAY-100635 maleate (WAY; Abcam, Cambridge, UK) (0.3 mg/kg) were dissolved in saline. FLX, MEM, and TAND were injected intraperitoneally (*i.p.*) 30 min before the behavior test. WAY was injected subcutaneously (*s.c.*) 50 min before the behavior test, and FLX was injected 20 min later. For the infusion of WAY (10.0μg/0.2 μl/mouse) to PFC, mice were anesthetized with three types of mixed anesthetics *i.e.* medetomidine hydrochloride (Domitol, Meiji Seika Pharma Co., Ltd., Tokyo, Japan), midazolam (Dormicum, Astellas Pharma Inc., Tokyo, Japan), and butorphanol (Vetorphale, Meiji Seika Pharma Co., Ltd.) (*i.p.*) and bilaterally implanted with a guide cannula (6 mm, 0.4 mm inner diameter, 0.5 mm outer diameter; Eicom, Kyoto, Japan) in the prefrontal cortex (coordinates: +1.7 mm anteroposterior, ±0.5 mm mediolateral from the bregma, -1.5mm dorsoventral from the skull). One day after recovery from surgery, mice were injected with WAY. WAY was dissolved in saline and (10.0μg/0.2 μl/mouse) were administered bilaterally into each prefrontal cortex through an infusion cannula that was connected to a microsyringe by a polyethylene tube. The entire infusion procedure took 2 min, and mice were handled gently to minimize stress. Immediately after infusion of WAY, FLX (20 mg/kg, *i.p.*) was administered. After 30 min of WAY infusion, mice were subjected to a sequence of behavioral tests (locomotor activity test, social interaction test, novel-object recognition test).

5. Real-time reverse transcription-PCR

The prefrontal cortex and dorsal raphe nucleus of each test mouse were homogenized, and total RNA was extracted using a RNeasy Total RNA Isolation Kit (Qiagen) and

converted into cDNA using a ReverTra Ace Kit for RT-PCR (Toyobo, Osaka, Japan). The primers used were as follows: for TPH2, forward primer, 5’-CATTCCTCGCACAATTCCAGTCG-3’, reverse primer, 5’-AGTCTACATCCATCCCAACTGCTG-3’; for SERT (Scl6a4), forward primer, 5’-AAGCCCCACCTTGACTCCTCC-3’, reverse primer, 5’-CTCCTTCCTCTCCTCACATATCC-3’; for MAO-A, forward primer, 5’-TCAATGTAGCCACTCCACTGT-3’, reverse primer, 5’-TTGGGGATAAAGTGAAGCTGA-3’; for β-actin, forward primer, 5’-CGATGCCCTGAGGCTCTTT-3’, reverse primer, 5’- TGGATGCCACAGGATTCCA-3’. PCRs were performed using the SsoAdvanced Universal SYBR Green SuperMix (Bio-Rad, Hercules, CA, USA). The reaction profile consisted of a first round at 95 °C for 30 sec and then 45 cycles of denaturation at 95 °C for 15 sec, annealing at 60 °C for 60 sec in a StepOne Real-Time PCR system (Life Technologies, Carlsbad, CA, USA). To standardize quantification, β-actin was calculated simultaneously with TPH2, SERT, and MAO-A. Expression levels were calculated by the delta-delta Ct method.

6. Western blotting analysis

Tissues were homogenized in ice-cold RIPA buffer (20 mM, pH-7.4 Tris-HCl, 150 mM NaCl, 1% NP-40, 2 mM EDTA, 1mM sodium ortho-vanadate, 50 mM NaF, 0.1% SDS and 1% sodium deoxycholate, 20 μg/ml pepstatin, 20 μg/ml aprotinin, and 20 μg/ml leupeptin) containing a Complete™, Mini Protease Inhibitor Cocktail (Roche Diagnostics, Mannheim, Germany) by sonication. After centrifugation at 15,000 rpm × 15 min × 4°C, the protein concentration in the supernatant was determined with Bradford (Millipore, Billerica, MA). Each protein sample was electrophoresed on 10% (w/v) SDS-PAGE and

subsequently transferred onto a 0.22 mm PVDF membrane (Millipore). The membrane was blocked with 5% skim milk in TBST for 60 min at room temperature and probed with a primary antibody at 4°C overnight. The PVDF membranes were washed three times for 5 min in TBST, incubated with the appropriate HRP-conjugated secondary antibody for 2 h at room temperature, and then washed three times for 5 min in TBST. Immunoreactive bands were visualized using an ATTO LuminoGraphI (ATTO, Tokyo, Japan). The band intensities were analyzed using CS Analysis 3.0 (ATTO). The membranes were stripped in stripping buffer (100 mM 2-mercaptoehanol, 2 % SDS, and 62.5 mM Tris-HCl, pH 6.7) at 55℃ for 30 min.

The primary antibodies were used as follows: rabbit anti-phospho-CaMKII α (Thr286) (1:1000, ab5683, Abcam), rabbit anti-CaMKII α (1:2000, C6974, Sigma-Aldrich St. Louis, MO, USA), rabbit anti-phospho-Akt (Ser473) (1:2000, #4060, Cell Signaling Technology), rabbit anti-Akt (pan) (1:1000, #4691, Cell Signaling Technology), rabbit anti-phospho-cAMP response element-binding protein (CREB) (Ser133) (1:1000, #9198, Cell Signaling Technology), mouse anti-CREB (1:1000, sc-377154, SANTA CRUZ Biotechnology. Ins, INC), rabbit anti-NMDAR1 (1:1000, ab109182, Abcam, Cambridge, UK), rabbit anti-phospho NR1 (Ser897) (1:1000, #ABN99, Millipore, Billerica, USA), rabbit anti-vesicular glutamate transporter (VGLUT1) (1:1000, #12331, Cell Signaling Technology), rabbit anti-EAAT2 (GLT1) (1:1000, #3838, Cell Signaling Technology), rabbit anti-PSD95 (1:1000, #2507, Cell Signaling Technology), rabbit anti-serotonin 1A (5-HT1A) receptor (1:500, ASR-021, Alomone labs, Jerusalem, Israel), and mouse anti-β-actin (1:1000, A5441, Sigma-Aldrich, St. Louis, MO).

7. Monoamine contents

Monoamine content was determined using an HPLC system (HTEC-500; Eicom). Each frozen brain sample was weighed and homogenized with an ultrasonic processor in 0.2M perchloric acid containing isoproterenol as an internal standard. The homogenates were placed on ice and centrifuged at 20,000 g for 15 min. The supernatants were mixed with 1 M sodium acetate to adjust the pH to 3.0 and injected into an HPLC system equipped with a reversed-phase ODS column (Eicompak SC-5DS; Eicom) and electrochemical detector.

8. *In vivo* micro dialysis

In vivo micro dialysis was performed as previously described [7]. Mice were anesthetized with three types of mixed anesthetic agents *i.e.* medetomidine hydrochloride (Domitol, Meiji Seika Pharma Co., Ltd., Tokyo, Japan), midazolam (Dormicum, Astellas Pharma Inc., Tokyo, Japan), and butorphanol (Vetorphale, Meiji Seika Pharma Co., Ltd.) (*i.p.*) before stereotaxic implantation of a guide cannula (AG-6; Eicom) into the prefrontal cortex (PFC) (+1.7 mm anteroposterior, +0.3 mm mediolateral, from bregma, -2.5 mm dorsoventral from the skull). One day after the operation, a micro dialysis probe (AI-4-2; 1 mm membrane length; Eicom) was inserted through the guide cannula and perfused with artificial CSF (147 mM NaCl, 4 mM KCl and 2.3 mM $CaCl_2$) at a flow rate of 1 μl/min. The dialysate was collected every 20 minutes and analyzed using HPLC with an electrochemical detector (HTEC-500; Eicom). Three time points were chosen to establish the baseline levels of extracellular neurotransmitters. To stimulate depolarization, Ringer’s solution containing 50 mM KCl was delivered through a dialysis probe for 10 min to measure the potassium-evoked release of glutamate, serotonin and GABA.

9. Opto-genetic manipulations

An optic fiber cannula (AGFL-4; Eicom) for micro dialysis and LED cannula (TeleLCT-B/R-4-250; Teleopto) for behavioral testing were placed targeting the dorsal raphe nucleus (DRN) (-4.5 mm anteroposterior, +0.3 mm mediolateral, from bregma, -2.25 mm dorsoventral from the skull). During micro dialysis, continuous blue light (470 nm), 5V light stimulation were repeated for 5 min. A wireless stimulation system (Teleopto, Bio Research Center, Japan) was used for opto-genetic behavioral experiments. Mice were habituated to a dummy receiver for 4 days before the behavioral tests. The stimulation device triggered light at 470 nm (blue light) with an intensity of 1.0 mW. The 10 Hz pulses were generated and spaced for 10 min at 2 s intervals. To measure the grooming frequency and duration, the mice were placed in an acrylic box for 1 h. The measurements were divided into two sessions of 30 min each. No light stimulation was performed during the first session. In the second session, light stimulation was administered for 5–30 min. The frequency and duration of grooming were compared between the light "on" and light "off" conditions. In the social interaction test, the mice were habituated to the apparatus for 2 days. On day 3, mice were placed with an unfamiliar partner in the apparatus for 10 min without light stimulation. On day 4, the mice were again placed with another unfamiliar partner in the apparatus with light stimulation. The duration of social behavior was compared between the light "on (day 3)" and light "off (day 4)" conditions. In the novel object recognition test, the mice were habituated to the apparatus for three days and subjected to a training session on day four and a retention session on day five without light stimulation. On day 6, they were subjected to a training session with light stimulation. On day 7, they were subjected to the retention session without light stimulation. The time spent exploring novel objects in the retention session was compared between the light

“on” and light “off” condition in the training session.

10. Immunofluorescence

Mice were anesthetized with chloral hydrate (40 mg/kg, *i.p.*) and perfused transcardially with 4% paraformaldehyde (PFA) in 0.2 M sodium phosphate buffer (pH 7.2). The brains were removed and kept in 4% PFA overnight at 4°C. The postfixed tissues were soaked in 20% (w/v) sucrose with PBS. The brains were embedded in OCT compound (Sakura Finetechnical Co., Tokyo, Japan), and cut into 20 μm sagittal sections using a cryostat (Leica CM3050, Wetzlar, Germany) for immunohistochemistry.

Cryosections were immuno-stained with rabbit anti-phospho-CaMKII α antibody (1:200, ab5683, Abcam, Cambridge, UK). The sagittal sections were autoclaved for 2 min (105℃) with 10 mM citrate buffer (pH 6.0) to activate antigens. After washing with PBS, sections were blocked with 5% fetal bovine serum (Nichirei Biscience Inc., Tokyo, Japan) in PBS containing 0.3% Triton-X (PBST) for 1h, then incubated with primary antibodies in PBS at 4°C overnight for 3 days. After washing with PBST, the sections were incubated with secondary antibodies (1:2000; A-11008, Alexa488-conjugated goat anti-rabbit IgG; Molecular Probes, Eugene, USA) and Hoechst 33342 (0.1 μg/ml; H342, Dojindo, Kumamoto, Japan) for 3 h at room temperature. Sections were mounted and covered with glass coverslips after rinsing with PBST and then visualized under a confocal laser microscope Zeiss LSM-710 (Carl Zeiss, Jena, Germany).

Supplemental results

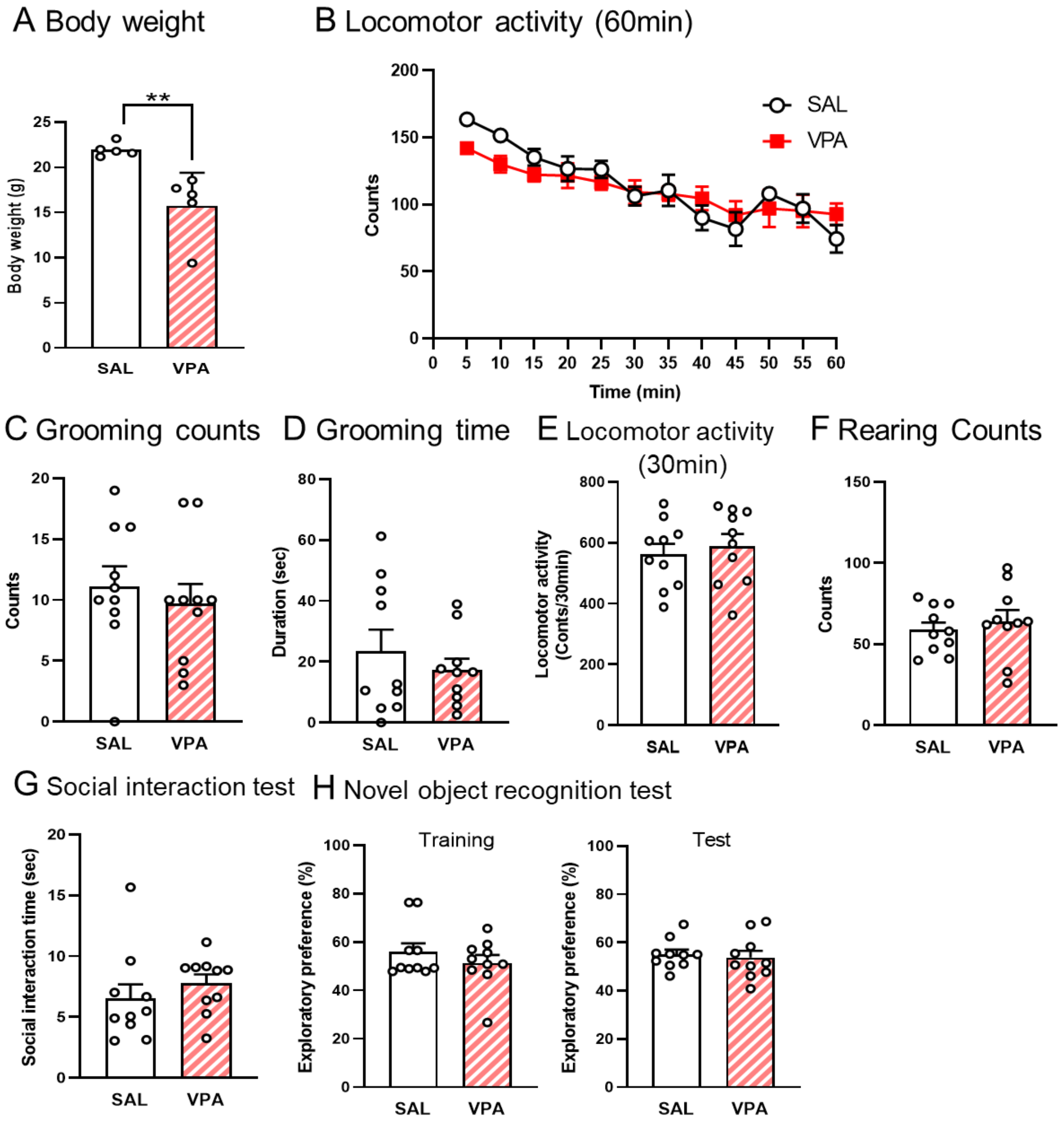

Supplemental Figure.1 Behavioral phenotype in the female prenatal VPA mice

Female prenatal VPA mice were measured body weight at 8 weeks old (A). Locomotor activity test (B-F): Locomotor activity was evaluated every 5 min for 60 min (B). Counting grooming behavior (C) and duration of grooming behavior (D) in the latter half of the test (31-60 min) for 30 min. Locomotor activity (E) and counting rearing behavior (F) in the latter half of the test. Social behavior in social interaction test (G)

and cognitive function in novel object recognition test (H) were evaluated. Each column represents the mean ± SEM. **$p<0.01$ versus control mice (SAL; saline). (A) n=5; (B-H) n=10/ each group.

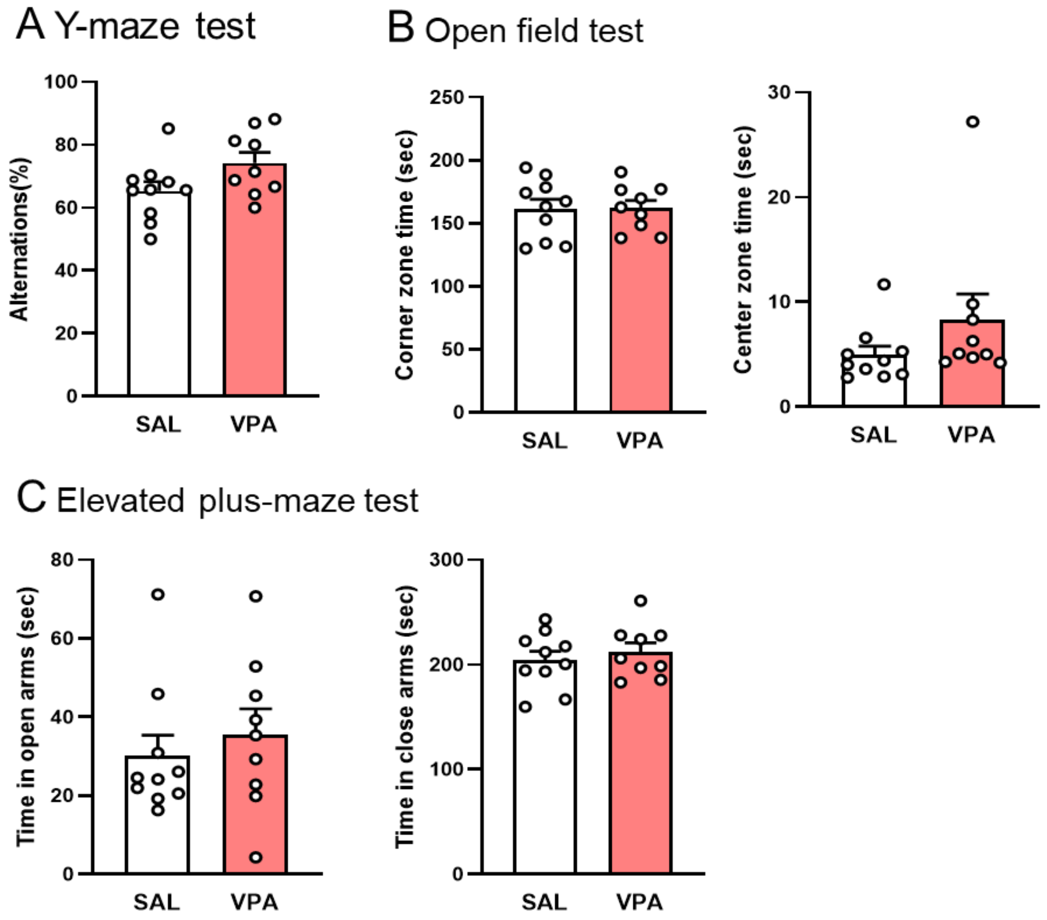

Supplemental Figure.2 Short-term memory and anxiety in the male prenatal VPA mice

(A) Short-term memory in Y-maze test: The alternation behavior was measured for 8 minutes. (B) Open field test: The prenatal VPA mice and SAL mice were placed in the open field, and the assessed the time spent in the corner and center areas for 5 minutes. (C) Elevated plus maze test: The prenatal VPA mice and SAL mice were placed in the elevated plus maze, and the assessed the time spent in the open arms and close arms for 5 minutes. Values represent the mean ± SEM. Each column represents the mean ± SEM. n=9-10/ each group.

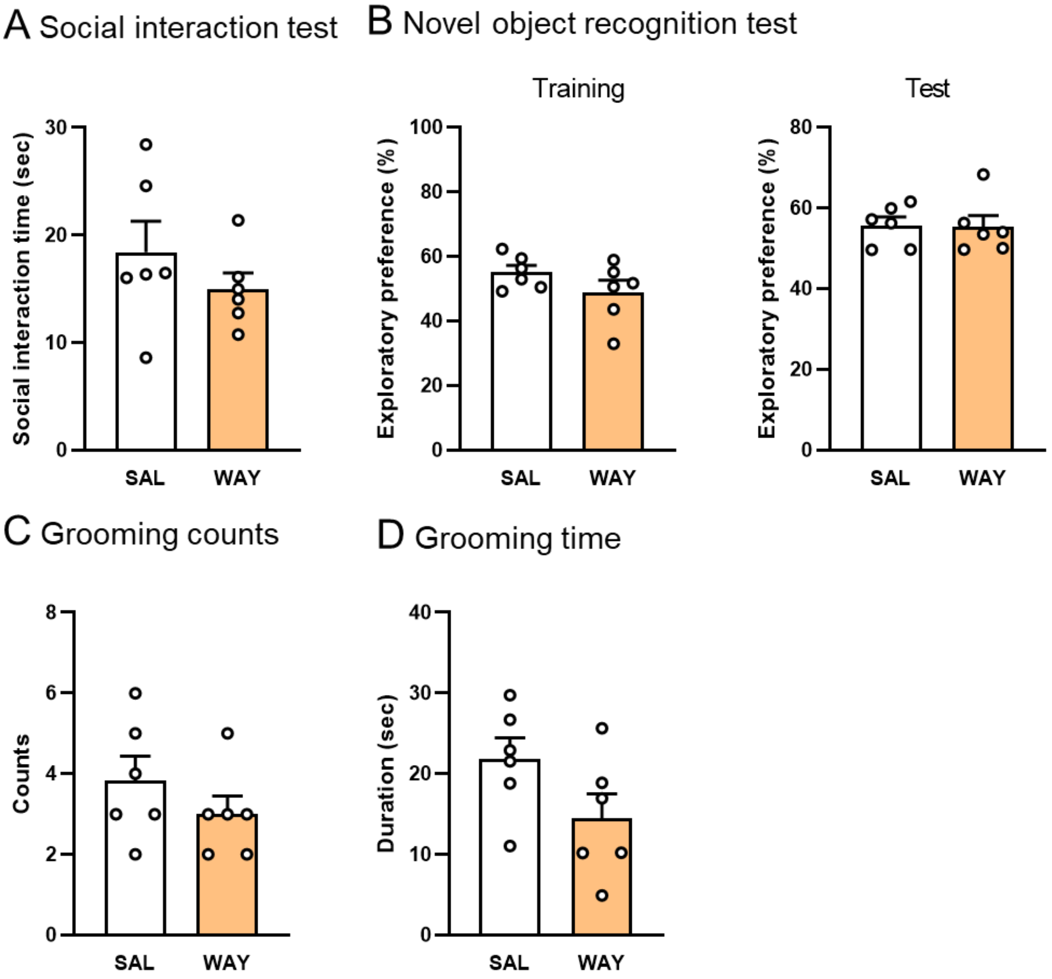

Supplemental Figure.3 Effects of WAY-100635 infusion into the prefrontal cortex on the emotional and cognitive function in the control mice

(A-D) Behavior tests after the WAY-100635 (WAY) infusion: Bilateral infusion of WAY (10.0μg/0.2 μl) into PFC was performed to control mice 30 min before social interaction test (A), novel object recognition test (B), and the measurement of counts (C) and duration (D) of grooming behavior. Each column represents the mean ± SEM. n=6/ each group.

Regions		Saline(nmol/mg tissue)	Valproic acid(nmol/mg tissue)
Prefrontal cortex	Dopamine	1093 ± 228.8	1076 ± 242.9
	DOPAC	764.7 ± 105.4	575.7 ± 71.65
	HVA	683.5 ± 73.11	571.0 ± 52.23
	5-HT	852.7 ± 28.77	825.1 ± 49.37
	5-HIAA	1767 ± 65.21	1500 ± 71.12 *
	NE	1016 ± 29.76	1075 ± 31.97
	MHPG	158.1 ± 40.32	250.4 ± 52.10
Striatum	Dopamine	35804 ± 3864	37600 ± 2950
	DOPAC	15598 ± 1765	16010 ± 1125
	HVA	8928 ± 832.5	9011 ± 585.0
	5-HT	1761 ± 145.2	1476 ± 84.82
	5-HIAA	3978 ± 243.8	3721 ± 194.7
	NE	681.8 ± 85.92	559.5 ± 63.61
	MHPG	706.2 ± 91.70	654.5 ± 85.01
Hippocampus	Dopamine	266.53 ± 36.31	240.27 ± 80.28
	DOPAC	237.2 ± 14.50	269.0 ± 19.72
	HVA	422.4 ± 13.16	401.7 ± 12.68
	5-HT	1328 ± 63.52	1285 ± 36.92
	5-HIAA	2756 ± 164.3	2374 ± 111.2
	NE	1071 ± 41.15	1107 ± 33.11
	MHPG	461.2 ± 64.30	356.1 ± 55.10
Amygdala	Dopamine	3220 ± 1511	1767 ± 133.8
	DOPAC	2866 ± 1355	1375 ± 64.25
	HVA	1704 ± 357.0	1218 ± 44.18
	5-HT	1456 ± 93.33	1415 ± 85.80
	5-HIAA	2861 ± 124.4	2693 ± 142.9
	NE	1128 ± 57.44	1185 ± 45.6
	MHPG	788.7 ± 62.90	686.4 ± 78.00

Supplementary Table.1 Monoamine metabolites in the brain

*p<0.05 versus control mice.

Reference

1 Miyazaki K, Miyazaki KW, Sivori G, Yamanaka A, Tanaka KF, Doya K. Serotonergic projections to the orbitofrontal and medial prefrontal cortices differentially modulate waiting for future rewards. Sci Adv. 2020;6(48).

2 Kataoka S, Takuma K, Hara Y, Maeda Y, Ago Y, Matsuda T. Autism-like behaviours with transient histone hyperacetylation in mice treated prenatally with valproic acid. Int J Neuropsychopharmacol. 2013;16(1):91-103.

3 Mouri A, Sasaki A, Watanabe K, Sogawa C, Kitayama S, Mamiya T, et al. MAGE-D1 regulates expression of depression-like behavior through serotonin transporter ubiquitylation. J Neurosci. 2012;32(13):4562-80.

4 Lu L, Mamiya T, Lu P, Niwa M, Mouri A, Zou LB, et al. The long-lasting effects of cross-fostering on the emotional behavior in ICR mice. Behav Brain Res. 2009;198(1):172-8.

5 Mouri A, Noda Y, Hara H, Mizoguchi H, Tabira T, Nabeshima T. Oral vaccination with a viral vector containing Abeta cDNA attenuates age-related Abeta accumulation and memory deficits without causing inflammation in a mouse Alzheimer model. FASEB J. 2007;21(9):2135-48.

6 Dodart JC, Mathis C, Ungerer A. Scopolamine-induced deficits in a two-trial object recognition task in mice. Neuroreport. 1997;8(5):1173-8.

7 Mouri A, Noda Y, Noda A, Nakamura T, Tokura T, Yura Y, et al. Involvement of a dysfunctional dopamine-D1/N-methyl-d-aspartate-NR1 and Ca2+/calmodulin-dependent protein kinase II pathway in the impairment of latent learning in a model of schizophrenia induced by phencyclidine. Mol Pharmacol. 2007;71(6):1598-609.
